# Endoscopic Thrombin Injection for Gastric Variceal Bleeding: A Systematic Review and Meta‐Analysis of Observational and Trial Data

**DOI:** 10.1002/deo2.70335

**Published:** 2026-04-27

**Authors:** Wen Xu, Da‐qin Zhan, Ruo‐lan Wang, Shu Jin, Hui‐guo Ding

**Affiliations:** ^1^ Department of Gastroenterology and Hepatology Beijing Youan Hospital Capital Medical University Beijing China; ^2^ Department of Gastroenterology Taihe Hospital Hubei University of Medicine Shiyan China; ^3^ Department of Pediatrics Taihe Hospital Hubei University of Medicine Shiyan China

**Keywords:** endoscopic cyanoacrylate injection, endoscopic treatment, gastric varices, single‐arm meta‐analysis, thrombin

## Abstract

**Objective:**

The safety of endoscopic thrombin injection (ETI) for treating the bleeding of gastric varix (GV) in patients with portal hypertension requires further evaluation. This meta‐analysis systematically reviews the available evidence on the efficacy and safety of ETI for GV bleeding.

**Methods:**

Two researchers independently screened and extracted data from all relevant original articles published from database inception to May 2025. Study quality was assessed using the Methodological Index for Non‐Randomized Studies tool. Meta‐analysis was performed using RevMan 5.3 software, with risk of bias assessed via risk of bias plots and funnel plots.

**Results:**

Thirteen studies involving 417 patients were included. Meta‐analysis revealed an initial hemostasis rate of 93% (95% confidence interval [*CI*]: 0.89–0.95), a 5‐day rebleeding rate of 11% (95% *CI*: 0.07–0.17), a late rebleeding rate of 14% (95% *CI*: 0.11–0.19), a complete GV obliteration rate of 36% (95% *CI*: 0.11–0.73), and a 6‐week GV‐related mortality rate of 9% (95% *CI*: 0.06–0.15). The overall complication rate was 2.2% (9/417), with fever and leukocytosis being the most common events.

**Conclusion:**

Endoscopic thrombin injection for GV bleeding appears to achieve high initial hemostasis with low rates of rebleeding in the available studies. Reported complication rates were low, though systematic assessment was not consistently described across studies. It is suggested the potential efficacy and an acceptable safety profile within the available evidences.

## 1 Introduction

A gastric varix (GV), which is composed of feeding vessels, variceal channels, and draining vessels, is a collection of veins in the stomach's submucosa and forms a complex network of vascular shunts between the portal and systemic circulation. Thus, endoscopically visible varices in the gastric mucosa are mere fractions of complicated and convoluted veins extending into the gastric serosa or beyond; these veins are characterized by intricate vascular anatomy and hemodynamics [1]. The prevalence of GVs in cirrhosis with portal hypertension ranges from 17% to 25%, versus 85% for esophageal varices. The incidence of GV ranges from 16% in 1 year to 44% in 5 years [2]. However, GV bleeding is usually severe, with 3‐year incidence rates of 16%–45%. After spontaneous hemostasis, over 35% of patients experience rebleeding, which is associated with a mortality rate as high as 45% and indicates persistently unsatisfactory clinical outcomes [3].

Recommended treatments for GV, including endoscopic cyanoacrylate injection (ECI), transjugular intrahepatic portosystemic shunt (TIPS), and balloon‐occluded retrograde transvenous obliteration (BRTO), remain controversial. ECI is the most commonly used, with initial hemostasis rates exceeding 90%. However, severe rebleeding occurs in 15%–20% of GV cases [4], and ECI has been linked to various complications, including glue‐induced endoscope obstruction, post‐injection ulceration, and severe ectopic embolism, which limit its broad application [5].

Endoscopic thrombin injection (ETI) has been explored as an alternative therapy for GV, in part due to its convenience of use and favorable safety profile. Unlike tissue adhesives, it does not damage the endoscope, and ectopic emboli and post‐injection ulcers are rare [5]. The British Society of Gastroenterology guidelines state that thrombin may also be considered for the treatment of bleeding GVs [6]. Oral thrombin was first used for upper gastrointestinal bleeding in 1947 [7], and it has since become widely used in endoscopic spray hemostasis. ETI for esophageal variceal bleeding was first reported in 1979 [8]. Thrombin serves as a hemostatic agent, converting fibrinogen into fibrin and increasing local platelet aggregation. Human thrombin has mostly replaced bovine thrombin due to concerns regarding prion transmission risk [9]. Published reports on ETI for GV have described high initial hemostasis rates, low rates of early and late rebleeding, and low 6‐week mortality, and do not cause serious adverse events [9, 10, 11, 12]. Therefore, we conducted this literature review and meta‐analysis to evaluate the efficacy and safety of ETI for bleeding GVs, aiming to provide evidence of its feasibility for clinical application.

## 2 Materials and Methods

### 2.1 Literature Search Strategy

Comprehensive computer searches were performed on PubMed, Web of Science, MEDLINE, The Cochrane Library, and ClinicalTrials.gov. Supplemental searches were conducted using Google Scholar. The search covers the time from the database's inception until May 2025, and a combination of Medical Subject Headings terms and free‐text keywords. The primary search strategy was as follows:

1. (“gastric varices” OR “gastric variceal” OR “gastric varix” OR “gastricesophageal varix” OR “gastricesophageal varices”)
2. (“bleeding” OR “hemorrhage” OR “haemorrhage” OR “rupture”)
3. (“endoscopic therapy” OR “endoscopic treatment” OR “endoscopic injection” OR “endoscopic management”)
4. (“thrombin” OR “human thrombin” OR “bovine thrombin” OR “recombinant thrombin” OR “fibrin glue” OR “fibrin sealant” OR “beriplast‐p”)
5. ① And ② AND ③ AND ④

### 2.2 Inclusion and Exclusion Criteria

Inclusion Criteria: ① Study Type: randomized controlled trials (RCTs), prospective or retrospective cohort studies, and other papers providing key outcome data; limited to English‐language publications; ② Study Subjects: human studies reporting ETI for GV bleeding; ③ Intervention: The experimental group primarily received ETI (via standard endoscopy or endoscopic ultrasound [EUS]) as the main treatment. The presence or absence of a control group was permitted; ④ Outcome Measures: studies reporting at least one of the following parameters: initial hemostasis rate, 5‐day rebleeding rate, late rebleeding rate, complete obliteration rate, complication rate, and 6‐week GV‐related mortality rate.

Exclusion Criteria: ① Studies where ETI was combined with sclerotherapy, coil embolization, TIPS, BRTO, or other primary therapies; ② Animal studies, single case reports, reviews, commentaries, conference abstracts, or papers lacking extractable key outcome data.

### 2.3 Clinical Terminology, Outcome Indicators, and Definitions

Clinical information recorded included acute bleeding status (active or recent), gastroesophageal varix (GOV) classification (GOV1/GOV2/IGV1/IGV2), and GV size (F1/F2/F3).

The primary outcomes were initial hemostasis rate, 5‐day rebleeding rate, late rebleeding rate, variceal complete obliteration rate, complications, and 6‐week GV‐related mortality.

The definitions were as follows:

1. Acute Variceal Bleeding: Encompassing both active bleeding at endoscopy (defined as visible spurting or oozing from a varices ) or and recent 5 days hematemesis or melena.
2. Recent Variceal Bleeding: Presence of one of the following signs during endoscopy: (i) GV with a white nipple sign or adherent clot; (ii) GV with red signs, blood in the stomach, and absence of other bleeding sources; (iii) GV with red signs, absence of blood in the stomach and clinical manifestations of hematemesis or melena, absence of other identifiable source [13].
3. GOV Classification: Based on Sarin's Classification: gastroesophageal varix type 1 (GOV1; extending along the lesser curvature); gastroesophageal varix type 2 (GOV2; extending into the gastric fundus); isolated GV type 1 (IGV1; located in the fundus); isolated GV type 2 (IGV2; located in the gastric body, antrum, pylorus, or duodenum).
4. GV Size: Classified using Hashizume's criteria [14]: F1 (diameter <5 mm; straight or mildly tortuous); F2 (5–10 mm diameter; nodular or tortuously thickened; with visible red signs, erosion, or thrombus cap); F3 (diameter >10 mm; tumorous, mass‐like, or complex network; with extensive erosion, ulceration, bluish discoloration, or active oozing).
5. Initial Hemostasis: Cessation of active bleeding observed endoscopically immediately after thrombin injection; further interventions (e.g., Sengstaken–Blakemore tube, additional endoscopic therapy, TIPS, or BRTO) are not required [15].
6. Five‐Day Rebleeding: Recurrence of bleeding within 5 days (120 h) after successful initial hemostasis, as confirmed by emergency endoscopy showing active bleeding or clinical manifestations of persistent hematemesis or melena. The consequences of recurrence are rare as follows: (a) hemodynamic instability; (b) need for blood transfusion; and (c) hemoglobin drop of >3 g/dL [13].
7. Late Variceal Rebleeding: Recurrence of bleeding more than 5 days and less 6 weeks after endoscopic treatment [13].
8. Complete Variceal Obliteration: Confirmed by follow‐up endoscopy showing hardened varices (resistant to catheter indentation) and reduction of ≤10 mm in diameter or by EUS 4 weeks or more after treatment; absence of intraluminal blood flow signals.
9. Complications: Fever (>38°C), post‐injection ulcer bleeding, bacterial infection, thromboembolism, or abdominal pain occurring after endoscopic treatment.
10. Six‐Week GV‐Related Mortality: Death attributable to GV bleeding within 6 weeks after endoscopic therapy [13].

### 2.4 Data Extraction and Quality Assessment

Two researchers separately screened the literature with the predetermined criteria and then cross‐checked the results. A third researcher reconciled the differences. Data were extracted using a standardized form, including study design, authors, country, publication year, sample size, thrombin type (bovine/human/beriplast), patient characteristics, etiology of portal hypertension, Child–Pugh score distribution, GOV type distribution, GV size distribution, thrombin concentration, total thrombin dose, number of injection sessions, outcomes (initial hemostasis, 5‐day rebleeding, late rebleeding, complete obliteration, complications, 6‐week GV‐related mortality), and follow‐up period.

Given that most of the included studies were retrospective and lacked control groups, the Methodological Index for Non‐Randomized Studies (MINORS) tool was adapted for quality assessment [16]. For the two included RCTs, we will assess the risk of bias using the Cochrane Risk of Bias tool for randomized trials (RoB 2), which is the current standard.

### 2.5 Statistical Analyses

RevMan 5.3 software was used for data synthesis and analysis. Heterogeneity among studies was assessed using the *I^2^* statistic. Given the predominance of retrospective studies and potential clinical heterogeneity, sensitivity analyses were performed using a random‐effects model for all outcomes to assess the robustness of the results. For single‐arm studies lacking a control group, the conversion method proposed by Chen et al. [17]. was utilized to pool event rates. The log odds ratio (*P*) was calculated as follows: *P* = ln(odds) = ln[*X*/(n–*X*)]. Standard error *SE(P)* = SE[In(odds)] =$\sqrt{1/X+1/(n-X)}\;$, where *X* is the number of *events* and n is the total sample size. The pooled odds ratio (*OR*) and its 95% confidence interval (*CI*) were then converted into the actual rate (*Pf*) and corresponding *CI* using the formulas *Pf* = *OR*/(1 + *OR*), upper limit (*UL*) = *UL_OR_*/(1 + *UL_OR_*), lower limit (*LL*) = *LL_OR_*/(1 + *LL_OR_*) (Table S1). This approach addresses the statistical challenge of pooling effect sizes in single‐arm studies.

## 3 Literature Screening Process and Results

**FIGURE 1:**
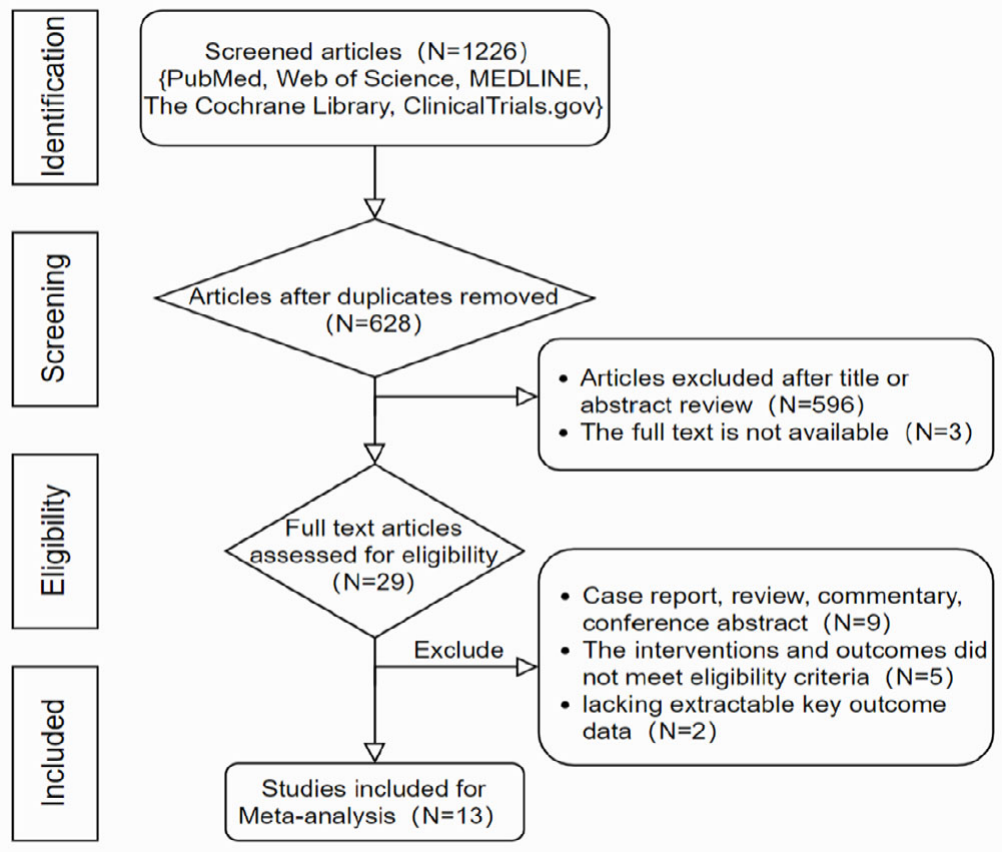
Flowchart of literature search and study selection.

The initial search yielded 1226 potentially relevant records. After duplicates were removed, 628 records remained. Screening titles and abstracts excluded 599 records. Three records were excluded due to full‐text unavailability or lack of valid data. The remaining 29 full‐text articles were assessed for eligibility. Sixteen articles were excluded for not meeting the inclusion criteria. Finally, 13 studies [9, 10, 11, 12, 18, 19, 20, 21, 22, 23, 24, 25, 26] were included in the meta‐analysis (Figure 1).

## 4 Basic Characteristics of Included Studies

The basic characteristics and MINORS scores of the included studies are summarized in Table S2. The 13 studies comprised two RCTs and 11 retrospective studies, examining 417 patients treated with ETI. Among them, 10 studies were conducted in the United Kingdom. Frost et al. [25] used EUS‐guided thrombin injection (EUS‐TI); the other studies used standard gastroscopy. Two studies (Datta et al. [22] and Heneghan et al. [20]) used Beriplast (a combination of lyophilized human thrombin, human fibrinogen, and diluent), three used bovine thrombin [10, 18, 19], and eight used human thrombin. Among the 417 patients, 359 presented with acute hemorrhage, two received primary prophylaxis, and 13 received secondary prophylaxis. Bleeding context was unspecified for the remaining 43. Child–Pugh scores were available for 347 patients: grades A (*n* = 91), B (*n* = 139), and C (*n* = 117). GOV classification was reported for 373 patients: GOV1 (*n* = 105), GOV2 (*n* = 195), IGV1 (*n* = 58), and IGV2 (*n* = 15).

MINORS quality assessment revealed insufficient reporting regarding adequate follow‐up time, prospective sample size estimation, and the presence of control groups in comparative studies. Total scores ranged from 14 to 21. The two RCT studies demonstrated low to moderate risk of bias by the use of RoB 2.

## 5 Meta‐Analysis Results

### 5.1 Initial Hemostasis

**FIGURE 2:**
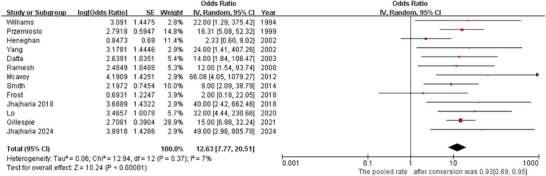
Forest plot for initial hemostasis rate.

All 13 studies reported initial hemostasis. Heterogeneity was low (*p* = 0.37, *I^2^* = 7%). ETI demonstrated a high initial hemostasis rate (*OR* = 12.63, 95% *CI*: 7.77–20.51; Figure 2). The pooled initial hemostasis rate was 93% (95% *CI*: 0.89–0.95).

### 5.2 Five‐Day Rebleeding

**FIGURE 3:**
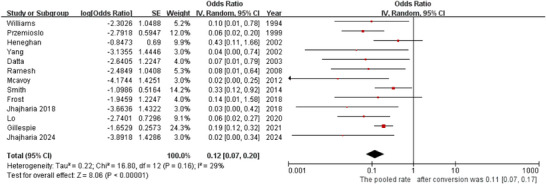
Forest plot for 5‐day rebleeding rate.

All 13 studies reported 5‐day rebleeding. Low‐to‐moderate heterogeneity was observed (*p* = 0.16, *I^2^* = 29%), warranting a random‐effects model. ETI was associated with a low 5‐day rebleeding rate (*OR* = 0.12, 95% *CI*: 0.07–0.20; Figure 3). The pooled 5‐day rebleeding rate was 11% (95% *CI*: 0.07–0.17).

### 5.3 Late Rebleeding

**FIGURE 4:**
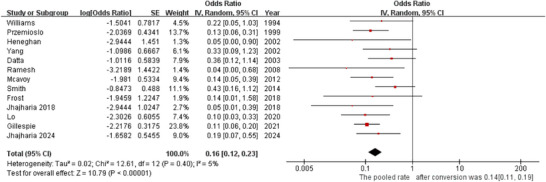
Forest plot for late rebleeding rate.

All studies reported late rebleeding. Heterogeneity was low (*p* = 0.40, *I^2^* = 5%). ETI was associated with a low late rebleeding rate (*OR* = 0.16, 95% *CI*: 0.12–0.23; Figure 4). The pooled late rebleeding rate was 14% (95% *CI*: 0.11–0.19).

### 5.4 Complete Obliteration

**FIGURE 5:**
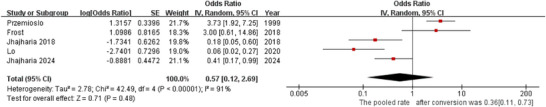
Forest plot for complete obliteration rate.

Only five studies reported rates of complete obliteration, as confirmed by follow‐up endoscopy or EUS. The meta‐analysis of these studies revealed a remarkable difference in the rate of complete obliteration (*OR* = 0.57, 95% *CI*: 0.12–2.69; Figure 5). The pooled complete obliteration rate was 36% (95% *CI*: 0.11–0.73). However, significant and considerable heterogeneity was observed (*p* < 0.001, *I^2^* = 91%), prompting the use of a random‐effects model and necessitating further investigation into its sources.

We investigated the potential sources of this heterogeneity. The included studies exhibited notable clinical diversity, particularly in (1) the method of assessment (one study used EUS while four used standard endoscopy) and (2) the timing of follow‐up (6–120 weeks), which are known to influence obliteration rates. This wide variation in study methodologies likely explains the high statistical heterogeneity and precludes a definitive conclusion from the pooled estimate.

### 5.5 Complications

Among the 417 patients, no post‐injection ulcers or severe complications (e.g., symptomatic ectopic embolism) were reported during follow‐up. Only two studies reported minor adverse events [12, 26], totaling nine events: abdominal pain (*n* = 1), urinary tract infection (*n* = 1), fever (*n* = 5), and leukocytosis (*n* = 2). The overall complication rate was 2.2% (9/417). However, adverse event reporting was not consistently described across all studies; only a subset of studies provided detailed systematic documentation.

### 5.6 Six‐Week GV‐Related Mortality

**FIGURE 6:**
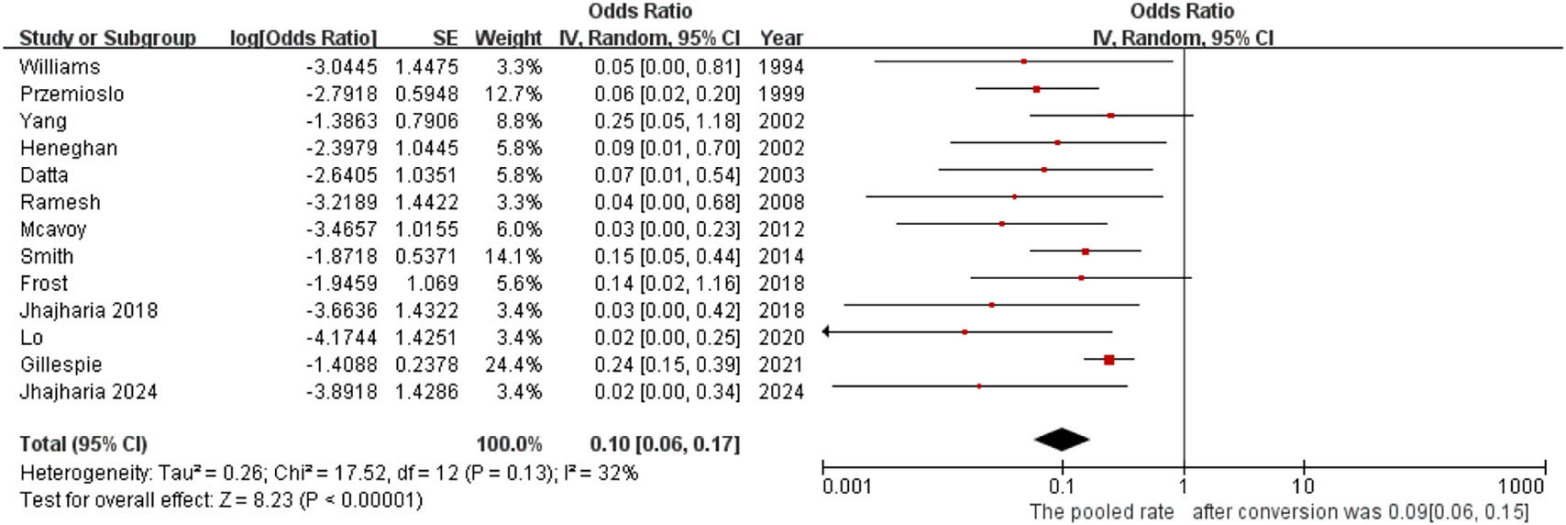
Forest plot for 6‐week gastric varix (GV)‐related mortality rate.

All studies reported 6‐week GV‐related mortality. Low‐to‐moderate heterogeneity was observed (*p* = 0.13, *I^2^* = 32%), supporting the use of a random‐effects model. ETI was associated with a low 6‐week GV‐related mortality rate (*OR* = 0.10, 95% *CI*: 0.06–0.17; Figure 6). The pooled mortality rate was 9% (95% *CI*: 0.06–0.15).

### 5.7 Evaluation of Publication Bias

**FIGURE 7:**
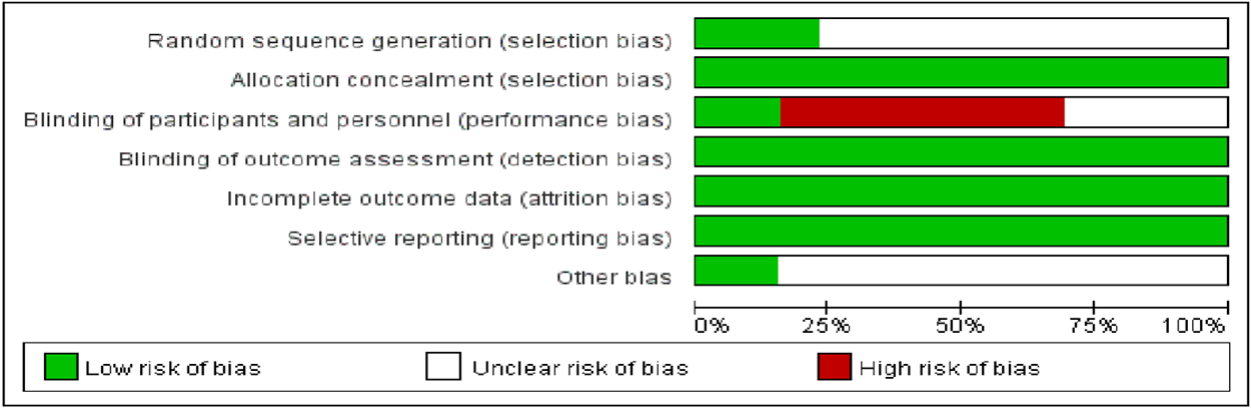
Risk of bias assessment plot.

**FIGURE 8:**
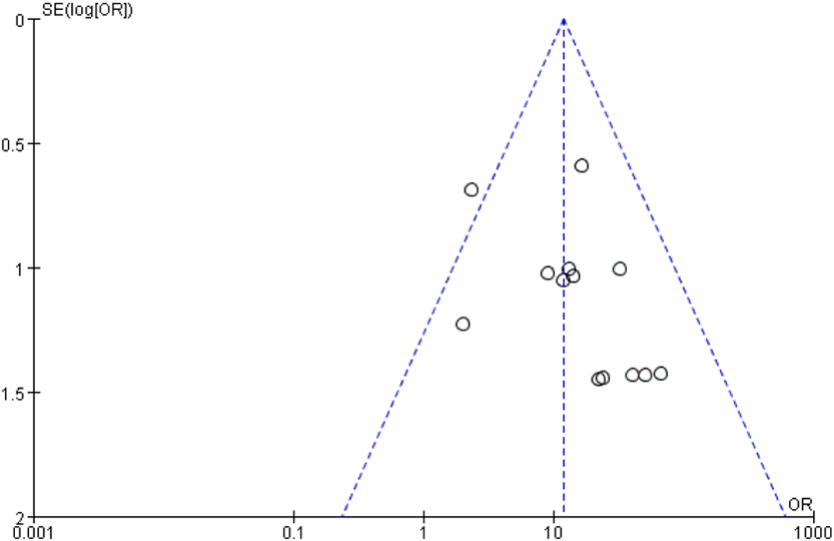
Funnel plot for initial hemostasis rate (assessment of publication bias).

Risk of bias assessment for the 13 studies is presented in a risk of bias plot (Figure 7). Potential reporting biases for the outcome of initial hemostasis were assessed using a funnel plot (Figure 8). The visual inspection revealed mild asymmetry, with a relative gap in the lower‐left region of the plot. This pattern is often suggestive of publication bias or other methodological factors. Given the limitation of a single‐arm meta‐analysis, formal statistical testing for asymmetry is not recommended due to its low power and unreliable interpretation. Therefore, visual assessment is presented for qualitative consideration only.

## 6 Discussion

GV bleeding is a severe complication of portal hypertension with high mortality, posing remarkable management challenges. Although ECI is widely used for GV bleeding, it carries a high risk of rebleeding and various complications, including ulceration in 65% of patients [12], bacteremia in 32% [27], symptomatic ectopic embolism in 4.4% [28], and imaging‐detected asymptomatic embolism in 47% [29]. Moreover, fatal distant embolization to the brain, lungs, and spleen has been reported after ECI [30]. These safety concerns highlight the continued interest in alternative GV therapies that are easy to perform, effective, and potentially associated with fewer complications.

Thrombin, as a hemostatic agent suitable for intravascular injection, has emerged as a promising alternative due to its simplicity and favorable safety profile. No cases of post‐injection ulcers or ectopic embolism have been reported to date [31]. It is widely used in interventional radiology as a liquid embolic agent, often alone or with coils, for arteriovenous fistulae and pseudoaneurysms [32]. It induces hemostasis by converting fibrinogen into fibrin and enhancing platelet aggregation. A 5 mL solution containing 5000 units of thrombin can clot 1 L of blood within 60 s. However, the efficacy of this solution depends on adequate fibrinogen levels and is thus ineffective for primary coagulopathy causing fibrinogen deficiency [33]. ETI has low rebleeding rates when used to treat esophageal, gastric, small intestinal varices, and peptic ulcer bleeding [9, 34].

Two recent RCTs comparing ETI and ECI for GV bleeding found comparable hemostasis rates but significantly lower complication rates with ETI. Lo et al. [12] randomized 33 patients to ETI and 35 to ECI. Initial hemostasis (90% vs. 90.9%, *p* = 0.58), 5‐day treatment failure (6.1% vs. 5.7%, *p* > 0.99), and 6‐week mortality (0% vs. 2.9%, *p* > 0.99) were comparable. However, the complication rate was significantly lower in the ETI group (12.1% vs. 51.4%, *p* < 0.001). Notably, two patients with post‐injection ulcer bleeding after ECI were successfully salvaged with ETI. Meanwhile, Jhajharia et al. randomized 25 patients to ETI and 27 to ECI. Initial hemostasis (100% vs. 87.5%, *p* = 0.44), 5‐day treatment failure (0% vs. 6.1%, *p* = 0.165), and 6‐week mortality (0% vs. 14.8%, *p* = 0.045) did not differ significantly, although mortality trended lower with ETI. Notably, post‐injection ulcer incidence was significantly lower with ETI (0% vs. 51.4%, *p* < 0.001), and two patients with variceal rebleeding after ECI were salvaged with ETI. Despite the small sample sizes, these studies suggest ETI offers hemostatic efficacy equivalent to that of ECI but has fewer complications and less post‐injection ulceration, providing endoscopists with a valuable new option.

Our meta‐analysis, encompassing 13 studies from diverse regions and 417 patients, summarizes the reported outcomes for ETI for GV bleeding. The cumulative initial hemostasis rate was 93% (95% *CI*: 0.89–0.95), 5‐day rebleeding rate 11% (95% *CI*: 0.07–0.17), late rebleeding rate 14% (95% *CI*: 0.11–0.19), and 6‐week GV‐related mortality rate 9% (95% *CI*: 0.06–0.15). For comparison, a current favored approach, EUS‐guided glue combined with coil embolization, reports an early rebleeding rate of 7% and mortality of 6.9% [35]. These outcomes appear comparable to those reported for other therapies, suggesting that thrombin may be an effective treatment option, and are consistent with the findings of the aforementioned RCTs [12, 26].

The low adverse event rate reported in association with thrombin is a notable finding of this analysis. Our analysis identified only nine minor adverse events (fever, *n* = 5; abdominal pain, *n* = 1; urinary tract infection, *n* = 1; leukocytosis, *n* = 2) among 417 patients (2.2% incidence). No serious adverse events (post‐injection ulcers, ectopic organ embolization, or fatalities) were reported. This favorable profile is likely attributable to thrombin's mechanism of action and short half‐life [31]. However, the low complication rate should be viewed in light of the heterogeneity in reporting practices. By contrast, a recent high‐quality meta‐analysis reported a 5.9% incidence of minor adverse events and 5.6% incidence of distant organ embolism with ECI [35], highlighting what appears to be a favorable safety profile in the included studies.

Another advantage of ETI is its simplicity. Once reconstituted, it can be injected through a standard variceal sclerotherapy needle without complex preparation. This feature is crucial in acute bleeding scenarios [18]. Furthermore, our analysis indicates satisfactory hemostasis can be achieved with a few injection sessions (mean 1.8 sessions; range of 1–9). McAvoy et al. [23] observed through long‐term follow‐up that rebleeding was rare after three endoscopic treatments. Jhajharia et al. [11] reported that only 4 of 20 patients required repeated thrombin injections. Therefore, achieving complete variceal obliteration through multiple injections may not be necessary for sustained hemostasis. It may be explained by the anatomy of GVs and the mechanism of thrombin‐induced hemostasis. GVs are complex vascular networks involving feeding vessels, a submucosal variceal plexus, and draining veins [1]. This creates a high‐flow, low‐resistance shunt between the portal and systemic circulations. ETI induces rapid, localized intraluminal thrombosis, which reduces flow and wall tension, thereby stabilizing the rupture site. This partial, flow‐controlling thrombus may achieve clinical quiescence even without angiographic obliteration.

Optimal dosing for ETI remains undefined. In the included studies, concentrations ranged from 100 to 1000 IU/mL (most commonly 200–500 IU/mL), and total doses ranged from 400 IU to 42,000 IU (500–5000 IU). Frost et al. [25] and Lo et al. [12] suggested that insufficient dosing increases rebleeding risk. Conversely, Smith et al. [24], comparing successful and failed treatments, found similar doses used (1000 IU vs. 1182 IU) and proposed that routine repeat injections 24–48 h after initial treatment reduce rebleeding and improve efficacy. Given these conflicting observations, controlled studies comparing different thrombin injection protocols with long‐term follow‐up are urgently needed to establish optimal concentration, dose, and injection frequency.

EUS‐TI may have an advantage over conventional endoscopic injection because it allows for the direct visualization of the variceal network, precise targeting, and real‐time assessment of treatment efficacy [25]. Frost et al. [25] performed EUS‐TI to treat patients with GV, achieving complete obliteration (defined as absence of intraluminal blood flow on EUS) in all patients during the initial session (mean dose of 4294 IU per patient). However, one patient showed recanalization on eight‐month follow‐up, possibly due to endogenous fibrinolysis, which degrades the thrombus over time. In our meta‐analysis, only five studies assessed complete obliteration, with a pooled rate of only 35%. This result is inconsistent with the low rebleeding rates. This discrepancy may stem from differing assessment methods, small sample sizes, or inadequate follow‐up durations. Utilizing EUS in the evaluation of thrombus formation and monitoring of treatment response can provide objective data for refining protocols [32].

The strengths of our meta‐analysis include clearly defined inclusion criteria and outcome measures based on the Baveno Consensus, with consistent findings and low heterogeneity. MINORS scores reflected good‐to‐excellent study quality. Although funnel plot asymmetry suggested possible publication bias, formal testing was precluded by the single‐arm design, warranting cautious interpretation of pooled estimates. Comprehensive patient‐level data (Child–Pugh, GOV type, and GV size) were detailed to contextualize outcomes in Table S2. This is the first systematic review dedicated solely to ETI for gastric variceal bleeding. We provide novel stratification and highlight key research gaps: optimization of treatment protocol, the role of EUS in ETI, long‐term efficacy and durability, and comparative trials.

In Japan and China, where ETI remains unapproved, established alternatives include ECI, TIPS, and BRTO. The findings of this international meta‐analysis represent a potential future option. Its technical simplicity and safety advantages might be particularly relevant in settings where glue‐related complications are a concern or as a bridging therapy. However, its introduction into the Japanese clinical algorithm would require local prospective studies to confirm efficacy and safety, formal health economic evaluations relative to existing standards, and subsequent regulatory review. Limitations include variability in concomitant therapies (Table S3), which may affect hemostasis and rebleeding rates. Most studies were retrospective and single‐arm, limiting evidence strength. High heterogeneity (*I* ^2^ = 95%) for obliteration outcomes persisted despite random‐effects modeling, likely due to inconsistent definitions and follow‐up. Future studies require standardized protocols and extended follow‐up.

In summary, available data from predominantly observational studies suggest that ETI may be a safe and technically feasible option for GV bleeding, with high initial hemostasis rates and low complication rates in reported series. However, the current evidence, consisting largely of observational data, is insufficient to recommend ETI as first‐line therapy. The technical simplicity of ETI and its minimal equipment requirements (standard endoscopy platform) enhance its accessibility in diverse healthcare settings. Yet, the availability and cost of thrombin preparations may still pose challenges in some regions with limited healthcare resources. ETI may be particularly useful in three scenarios: (1) as a bridge to definitive therapy (TIPS, BRTO, and liver transplantation); (2) as a treatment for secondary prophylaxis; and (3) as an alternative for patients unsuitable for TIPS or BRTO. Large‐scale RCTs are warranted to further validate these findings and define ETI's precise role in the GV management algorithm.

## Author Contributions

We employed artificial intelligence tools to assist with language refinement during the revision process, enhancing the clarity and fluency of the manuscript. Wen Xu and Hui‐guo Ding conceived and designed this study; Wen Xu and Da‐qin Zhan searched for and collected the data; Ruo‐lan Wang and Shu Jin performed the statistical analysis and interpretation of data; Wen Xu and Da‐qin Zhan wrote the manuscript. Hui‐guo Ding contributed to funding acquisition and editing. All authors read and approved the final manuscript.

## Conflicts of Interest

The authors declare no conflicts of interest.

## Funding

This study was supported by the Beijing Municipal Science & Technology Commission (Z221100007422002).

## Supporting information




**TABLE S1**: Date of meta‐analysis.

**TABLE S2**: Basic characteristics of the included studies.

**TABLE S3**: Summary of concomitant therapies in included studies.
